# The biological role of extracellular vesicles in gastric cancer metastasis

**DOI:** 10.3389/fcell.2024.1323348

**Published:** 2024-01-25

**Authors:** Yun Lei, Shuang Cai, Chun-Dong Zhang, Yong-Shuang Li

**Affiliations:** ^1^ Department of Surgical Oncology and 8th General Surgery, The Fourth Affiliated Hospital of China Medical University, Shenyang, Liaoning, China; ^2^ Department of Gastroenterology, The Fourth Affiliated Hospital of China Medical University, Shenyang, Liaoning, China

**Keywords:** gastric cancer, extracellular vesicles, metastasis, tumor microenvironment, epithelial-mesenchymal transition

## Abstract

Gastric cancer (GC) is a tumor characterized by high incidence and mortality, with metastasis being the primary cause of poor prognosis. Extracellular vesicles (EVs) are an important intercellular communication medium. They contain bioactive substances such as proteins, nucleic acids, and lipids. EVs play a crucial biological role in the process of GC metastasis. Through mechanisms such as remodeling the tumor microenvironment (TME), immune suppression, promoting angiogenesis, and facilitating epithelial–mesenchymal transition (EMT) and mesothelial–mesenchymal transition (MMT), EVs promote invasion and metastasis in GC. Further exploration of the biological roles of EVs will contribute to our understanding of the mechanisms underlying GC metastasis and may provide novel targets and strategies for the diagnosis and treatment of GC. In this review, we summarize the mechanisms by which EVs influence GC metastasis from four aspects: remodeling the TME, modulating the immune system, influencing angiogenesis, and modulating the processes of EMT and MMT. Finally, we briefly summarized the organotropism of GC metastasis as well as the potential and limitations of EVs in GC.

## 1 Introduction

Gastric cancer (GC) is one of the most prevalent tumors globally. According to the 2020 Global Cancer Epidemiology report, GC has the fifth highest incidence and the fourth highest mortality (Sung et al., 2021). Metastasis is the leading cause of death in GC patients, with more than 90% of GC-related deaths attributed to metastasis. Patients with GC metastasis often survive less than 1 year (Orditura et al., 2014). Tumor metastasis follows the theory of “seed” and “soil,” being influenced by both the characteristics of the primary tumor cells and the microenvironment of the metastatic site (Akhtar et al., 2019). The tumor microenvironment (TME) refers to the complex environment composed of cells, stroma, and molecular components surrounding the tumor, it plays an important role in the process of GC metastasis, promoting tumor cell dissemination through various mechanisms such as immune response modulation, extracellular matrix remodeling, and angiogenesis promotion (Tang et al., 2022). Epithelial-mesenchymal transition (EMT) refers to the process in which epithelial cells lose their epithelial characteristics and acquire enhanced mesenchymal features, resulting in a more invasive and migratory phenotype. Similarly, mesothelial-mesenchymal transition (MMT) refers to the process in which mesothelial cells undergo changes in cell morphology and acquire characteristics of mesenchymal cells. Both types of cellular transformation enhance the invasive and metastatic capabilities of tumor cells, thereby promoting tumor metastasis (Sandoval et al., 2013; Li et al., 2023a). Tumor cell metastasis is also influenced by the immune system. Under normal circumstances, tumor cells can be recognized and eliminated by the immune system, inhibiting tumor development. However, tumor cells can evade immune system attacks through various mechanisms, thereby promoting tumor metastasis (Becerril-Rico et al., 2021). Additionally, adequate nutrient supply is essential for tumor growth and metastasis. Neoangiogenesis provides a foundation for tumor growth and metastasis, while vascular leakage provides more pathways for tumor cell dissemination (Wang et al., 2022a). These factors interact with each other, ultimately promoting GC metastasis.

Extracellular vesicles (EVs) are membrane-enclosed vesicles released by cells, and they can be secreted by almost all cell types. Their diameters typically range from 30 to 5,000 nm. Generally, based on the size, origin, and functional characteristics of EVs, they can be classified into different subtypes, including exosomes, microvesicles, and apoptotic bodies (Kalluri and McAndrews, 2023; Pourali et al., 2023). EVs were discovered as early as the 1960s, but at that time, little was known about them (Bonucci, 1967; Wolf, 1967; Anderson, 1969). Subsequent research has shown that biological substances such as nucleic acids, proteins, and lipids are found in EVs; they can interact with recipient cells, transmit information, and alter the function and behavior of recipient cells, playing a crucial role in intercellular communication (Majood et al., 2022; Kalluri and McAndrews, 2023).

**FIGURE 1:**
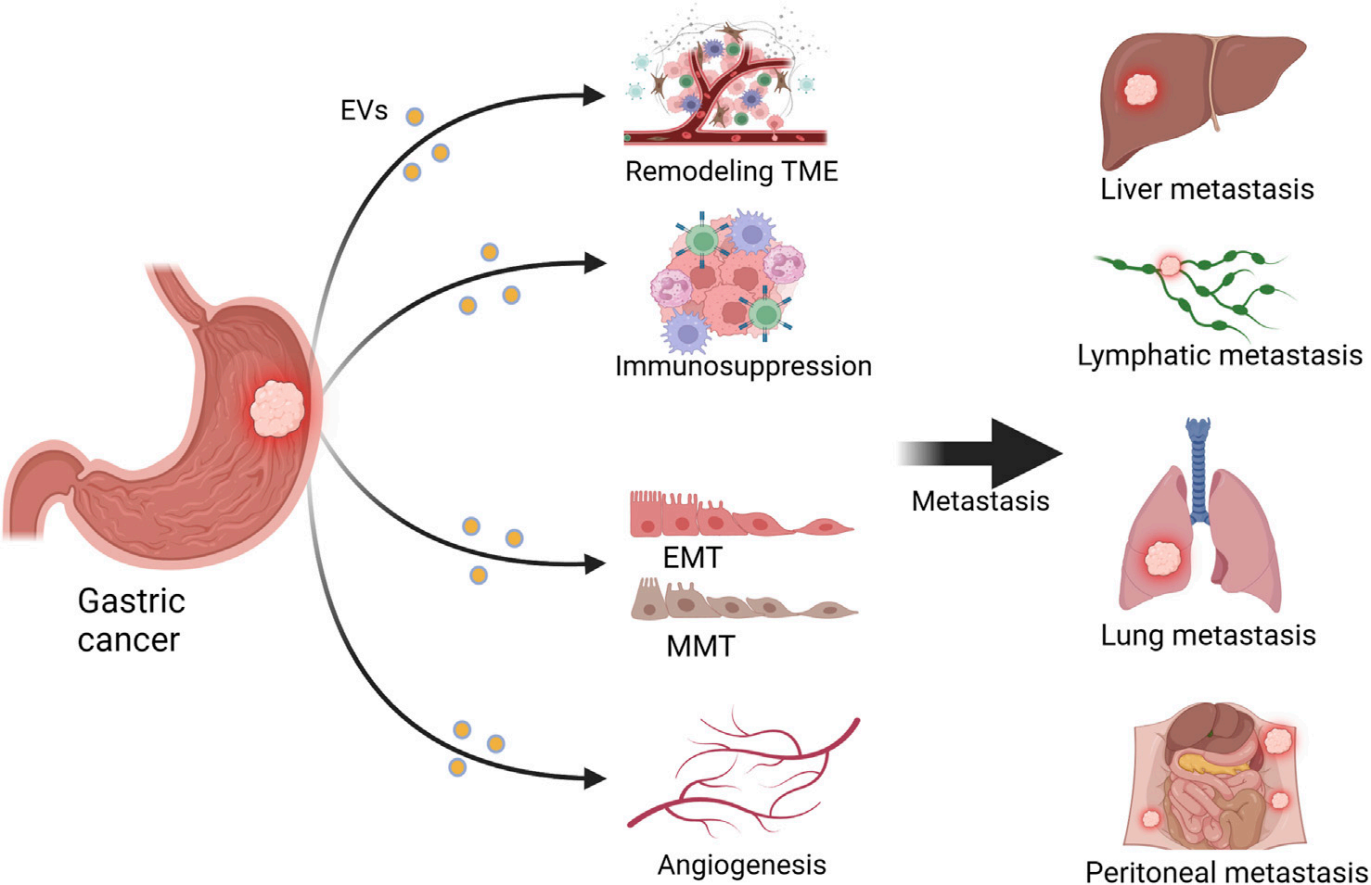
Extracellular vesicles (EVs) influence gastric cancer (GC) metastasis. EVs can promote GC metastasis to sites such as the liver, lungs, lymph nodes, and peritoneum by remodeling the tumor microenvironment (TME), inhibiting the immune system, promoting angiogenesis, and facilitating epithelial–mesenchymal transition (EMT) and mesothelial–mesenchymal transition (MMT). Figure was created with BioRender.com.

Numerous studies have reported a close association between EVs and GC metastasis. EVs exhibit a dual role in GC metastasis, as they can both promote and inhibit GC metastasis. This may depend on factors such as the cargos, origins, and characteristics of the recipient cells of EVs (Kalluri and McAndrews, 2023). EVs can influence GC metastasis through remodeling the TME, modulating the immune system, influencing angiogenesis, and modulating the processes of EMT and MMT (Gao et al., 2021a). Figure 1 and Table 1 summarize the relevant mechanisms by which EVs influence GC metastasis. Previous studies have summarized the role of EVs in promoting the formation of pre-metastatic niches in GC, whereas this review focuses on systematically and comprehensively summarizing the bidirectional regulatory role of EVs in GC metastasis from the above-mentioned aspects, expanding our understanding of the role of EVs in GC metastasis. The clinical translation of this knowledge will be helpful for the early treatment of GC.

**TABLE 1:** Mechanisms by which extracellular vesicles influence gastric cancer metastasis.

EVs cargos	Targets	Mechanism	Roles	Refs
miR-27a	SGC7901	Downregulate CSRP2	Promote metastasis	Wang et al. (2018)
Circ_0088300	SGC7901, BGC823	Target miR-1305/JAK/STAT axis	Promote metastasis	Shi et al. (2021)
lncRNA DACT3-AS1	HGC-27	Target miR-181a-5p/sirtuin 1 axis	Inhibit metastasis	Qu et al. (2023)
miR-139	HGC-27	Reduce the expression of MMP11 in the TME	Inhibit metastasis	Xu et al. (2019)
CD9	OCUM-12, NUGC-3	-	Promote metastasis	Miki et al. (2018)
miR-301b-3p	SGC-7901	Inhibit TXNIP	Promote metastasis	Zhu et al. (2022a)
THBS2	MGC-803 and mice	-	Promote metastasis	Qi et al. (2023)
miR-221	BGC-823, HGC-27, SGC-7901	-	Promote metastasis	Wang et al. (2014), Ma et al. (2017)
miR-29b	MKN-45, NUGC-4, Murine model	Inhibit TGF-β1 to suppress MMT	Inhibit metastasis	Kimura et al. (2023)
miR-374a-5p	AGS, HGC-27	Upregulate the expression of adhesion molecules in GC cells by targeting HAPLN1	Promote metastasis	Ji et al. (2023)
-	HGC-27	Inducing EMT by activating the AKT signaling pathway	Promote metastasis	Gu et al. (2016)
CD44	AGS, HGC-27	Activate of ERK/PPARγ/CPT1A pathway increases FAO activity in BM-MSCs, resulting in the secretion of IL-8 and STC1	Promote metastasis	Huang et al. (2023b)
LINC01559	AGS, HGC-27	Sponge miR-1343-3p to upregulate PGK1 and downregulate PTEN, therefore activate PI3K/AKT pathway	Promote metastasis	Wang et al. (2020)
UBR2	P53mBMMSC, MFC	Regulate Wnt/β-catenin pathway	Promote metastasis	Mao et al. (2017)
L-PGDS	SGC-7901, murine model	Reduce the expression of stem cell markers, inhibit STAT3 phosphorylation	Promote metastasis	You et al. (2022)
HMGB1	OCUM‐1 and MGC‐803	Induction of M2-like polarization of macrophages by inhibiting the NF-κB pathway	Promote metastasis	Liu et al. (2023)
ELFN1-AS1	THP-1	Induce M2 polarization of macrophages by ELFN1-AS1/miR-4644/PKM axis	Promote metastasis	Ma et al. (2023a)
-	M0-M, M0-GM	Induce M2 polarization of macrophages via the STAT3 pathway	Promote metastasis	Ito et al. (2021)
miR4435-2HG	MKN-45, AGS, mice	Promote M2 polarization of macrophages by regulating Jagged1/Notch and JAK1/STAT3 axes	Promote metastasis	Li et al. (2022)
miR-519a-3p	THP-1, mice	Activate the MAPK/ERK pathway by targeting DUSP2, thereby causing M2-like polarization of macrophages	Promote metastasis	Qiu et al. (2022)
miR‐92a‐3p	BMDM	Induce macrophage PD‐L1 expression by activating ERK signaling via inhibiting PTEN expression in BMDM	Promote metastasis	Gu et al. (2023)
hsa_circ_0017252	THP-1, mice	Inhibit macrophage M2 polarization by sponging miR-17-5p	Inhibit metastasis	Song et al. (2022)
-	AGS, HGC27	Activate the P38MAPK pathway and upregulate the expression of PD-L1	Promote metastasis	Wang et al. (2021a)
ApoE	MGC-803, MFC, mice	Activate PI3K/AKT/mTOR signaling pathway	Promote metastasis	Zheng et al. (2018)
TGF-β1	Naïve T cells	Induce naïve T cells’ transition to FOXP3 regulatory T cells that mediate immunosuppressive effect	Promote metastasis	Yen et al. (2017)
-	CD8 T cell, mice	Increases frequency of effector memory CD4 T and MDSC, decreases CD8 T cell and NK frequency, developing an immunosuppressive TME	Promote metastasis	Liu et al. (2020)
-	Neutrophils	Induce autophagy and pro-tumor activation of neutrophils via HMGB1/TLR4/NF-κB signaling	Promote metastasis	Zhang et al. (2018a)
miR-1246	Mice, AGS, HGC-27	Promote PD-L1 expression and CD8^+^ T cell apoptosis by downregulating GSK3β	Promote metastasis	Lu et al. (2023)
miR4435-2HG	MKN-45, AGS, mice	Promote M2 polarization of macrophages by regulating Jagged1/Notch and JAK1/STAT3 axes, promote EMT	Promote metastasis	Li et al. (2022)
miR-552-5p	SGC-7901, AGS, mice	Promote EMT by interfering with the PTEN/TOB1 axis	Promote metastasis	Zhu et al. (2022b)
miR-223	SGC-7901	Induce EMT by targeting the PTEN-PI3K/AKT pathway	Promote metastasis	Zheng et al. (2020)
LncRNA HOTAIR	NCI-N87, MKN45	Regulate the expression of EMT-related proteins	Promote metastasis	Chen et al. (2023b)
LncRNA ZFAS1	MKN28	-	Promote metastasis	Pan et al. (2017)
LncRNA PCGEM1	AGS, MKN45	Maintain stability and reduce the degradation of SNAI1, which could promote the EMT	Promote metastasis	Piao et al. (2021)
LINC01480	AGS, NCI-N87 and mice	Upregulation of VCAM1 expression promotes EMT by binding to miR-204-5p	Promote metastasis	Zhang et al. (2023a)
FRLnc1	HGC-27, MKN45	Activate ERK signaling pathway to promote the EMT	Promote metastasis	Zhang et al. (2021a)
LINC00355	AGS	Interact with HDAC3 to suppress TP53INP1 transcription, which promotes EMT	Promote metastasis	Zhao et al. (2023)
miR-196, miR92, miR1307	HGC-27, AGS, mice	Induce EMT by promoting the expression of EMT-related proteins	Promote metastasis	Hu et al. (2019)
miR-423-5p	SGC-7901, HGC-27	Suppress the expression of the SUFU to promote EMT	Promote metastasis	Yang et al. (2018)
miR-301a-3p	MGC-803, MKN-45	Promote EMT via MiR-301a-3p/PHD3/HIF-1α positive feedback loop	Promote metastasis	Xia et al. (2020)
TRIM3	MGC-803, SGC-7901, mice	Downregulate the EMT regulators to induce the EMT	Inhibit metastasis	Fu et al. (2018)
GKN1	AGS, MKN1, tumor tissue	Inhibit EMT by regulating EMT-related protein expression	Inhibit metastasis	Yoon et al. (2020)
miR-486-5p	HMrSV5	Inhibit EMT by downregulating SAMD2, CDK4 and ACTR3	Inhibit metastasis	Lin et al. (2021)
circ-ITCH	HGC-27, MGC-803, MKN-45	Inhibit EMT via regulating circ-ITCH/miR-199a-5p/Klotho axis	Inhibit metastasis	Wang et al. (2021b)
MMP2	MGC-803, HMrSV5	Activate the MAPK/ERK signaling to induce MMT	Promote metastasis	Deng et al. (2017)
miR-106a	HMrSV5, abdominal tumor model	Induce MMT via targeting Smad7	Promote metastasis	Zhu et al. (2022c)
miR-21-5p	HMrSV5, mice	Induce MMT by activating TGF-β/Smad pathway via target SMAD7	Promote metastasis	Li et al. (2018)
SNHG2	AGS, mice	Sponge miR-129-5p to boost E2F7 expression and activate the MAPK/ERK signaling, thus induce MMT	Promote metastasis	Zhang et al. (2022a)
miR-519a-3p	HUVEC	Activate the MAPK/ERK pathway by targeting DUSP2, thereby inducing angiogenesis	Promote metastasis	Qiu et al. (2022)
miR-29a/c	HUVEC, mice	Inhibit angiogenesis by suppressing VEGF expression	Inhibit metastasis	Zhang et al. (2016)
GRP78	HUEhT-1cells	Induce angiogenesis by increasing AKT phosphorylation	Promote metastasis	Iha et al. (2022)
circ29	HUVEC	Induce angiogenesis by sponging miR-29a to regulate the VEGF pathway	Promote metastasis	Li et al. (2021)
ANG2	HUVECs, mice	Induce angiogenesis by activating the PI3K/Akt signal pathway	Promote metastasis	Kalfon et al. (2022)
circ-RanGAP1	HGC-27	Sponge miR-877-3p to upregulate VEGFA expression	Promote metastasis	Lu et al. (2020)
circSHKBP1	BGC823, HGC27, mice	Regulate the miR-582-3p/HUR/VEGF axis	Promote metastasis	Xie et al. (2020)
circFCHO2	HGC-27, AGS	Activate the JAK1/STAT3 pathway via sponging miR-194-5p to induce angiogenesis	Promote metastasis	Zhang et al. (2022b)
HGF siRNA	HUVEC, mice	Inhibit angiogenesis by suppressing HGF/VEGF expression	Inhibit metastasis	Zhang et al. (2018b)
-	HUVEC, mice	Disrupt the endothelial barrier to promote the transendothelial migration of tumor cells	Promote metastasis	Wang et al. (2022b)

Abbreviations: TME, tumor microenvironment; MMT, mesothelial–mesenchymal transition; GC, gastric cancer; EMT, epithelial–mesenchymal transition.

## 2 EVs affect the metastasis of GC by remodeling the TME

**FIGURE 2:**
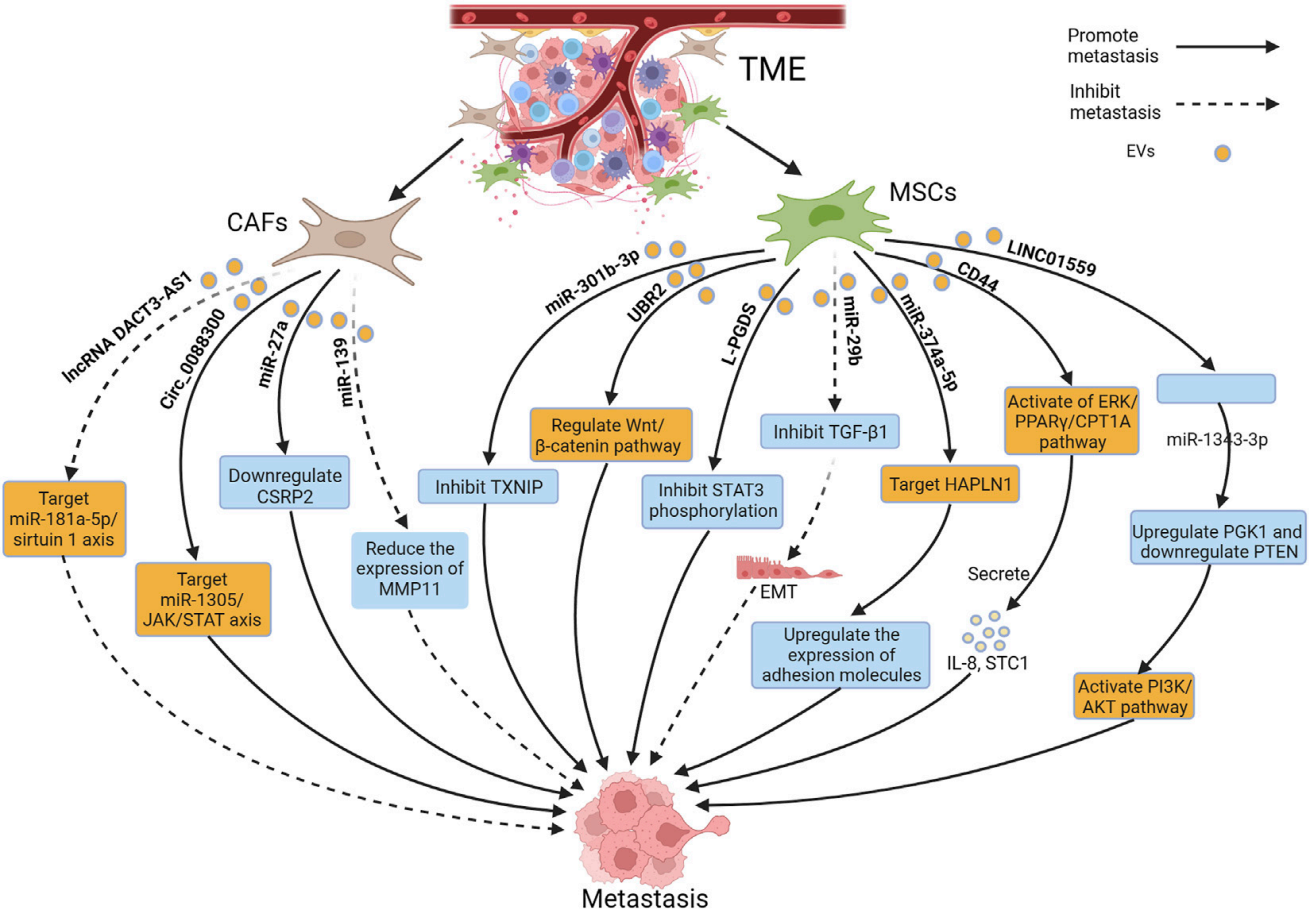
Extracellular vesicles (EVs) derived from cancer-associated fibroblasts (CAFs) and mesenchymal stem cells (MSCs) in the tumor microenvironment (TME) regulate gastric cancer (GC) metastasis through relevant mechanisms. Figure was created with BioRender.com.

The TME refers to the complex ecosystem composed of cells and the extracellular matrix surrounding the tumor. It includes mesenchymal stem cells (MSCs), immune cells, cancer-associated fibroblasts (CAFs), the extracellular matrix, and EVs, among others (Majood et al., 2022). The TME plays a crucial role in GC metastasis, where processes like angiogenesis, immune evasion, tumor-related inflammation, and matrix remodeling interact to promote the dissemination of GC cells (Yang et al., 2023). Numerous studies have reported that EVs modulate the TME through various pathways, influencing the invasive and migratory abilities of tumor cells and ultimately leading to tumor metastasis. Among these, EVs derived from CAFs and MSCs exhibit important regulatory effects on the TME. Figure 2 depicts the relevant mechanisms by which EVs remodel the TME to influence GC metastasis.

### 2.1 The impact of CAF-derived EVs on the TME

CAFs are important components of the TME and can originate from various precursors, including normal fibroblasts, MSCs, and pericytes (Ning et al., 2018). CAFs possess multiple cellular functions, including extracellular matrix deposition, metabolic reprogramming, and chemoresistance, therefore, they play a significant role in tumor metastasis (LeBleu and Kalluri, 2018; Huang et al., 2023a). The oncogenic gene, miR-27a, can induce fibroblast reprogramming into CAFs, and is overexpressed in GC-derived EVs. Additionally, overexpression of miR-27a in CAFs can enhance the metastatic behavior of GC cells by downregulating CSRP2, thereby assisting tumor cells both *in vitro* and *in vivo* during migration and invasion, but its downstream mechanisms are still unclear (Wang et al., 2018). Human GC tissues and plasma significantly overexpress circ_0088300 and circ_0088300 from CAF-derived EVs sponged to miR-1305, leading to downregulation of the JAK/STAT pathway and facilitating tumor metastasis in GC cells (Shi et al., 2021). However, Qu et al. reported that CAF-derived EVs carrying the disheveled binding antagonist of beta catenin3 antisense1 were found to suppress the malignant transformation of GC cells, including migration and invasion, by targeting the miR-181a-5p/sirtuin 1 axis. Additionally, they increased the sensitivity of GC cells to oxaliplatin (Qu et al., 2023). Similarly, Xu et al. reported that EVs derived from CAFs carried miR-139, which suppressed the metastasis of GC by reducing the expression of matrix metalloproteinase 11, both *in vivo* and *in vitro* within the TME (Xu et al., 2019). In addition, EVs derived from CD9-positive CAFs significantly stimulated the metastasis of scirrhous-type GC cells, and CD9-positive GC patients had significantly lower 5-year survival, when compared with CD9-negative GC patients. However, the specific molecular mechanisms by which these EVs promote GC cell metastasis are not yet fully understood (Miki et al., 2018).

### 2.2 The impact of MSC-derived EVs on the TME

MSCs are another important component of the TME, and play a crucial regulatory role in the occurrence and development of tumors, involving anti-cancer effects, regulation of angiogenesis, and anti-apoptosis functions (Kolf et al., 2007; Kidd et al., 2009; Ho et al., 2013; Li et al., 2020a). EVs have also been recently found to play a crucial role in tumor metastasis, particularly EVs derived from MSCs, which have gained increasing attention (Zhao et al., 2021; Zhu et al., 2022a; Qi et al., 2023). The overexpression of miR-221 is significantly associated with advanced tumor lymphatic metastasis (Liu et al., 2012), and a study reported that MSC-derived EVs delivered miR-221 to GC cells and promoted their metastasis (Wang et al., 2014). Several studies have also reported that MSC-derived EVs can effectively influence the TME and tumor metastasis through the delivery of miRNAs (Ma et al., 2017; Ji et al., 2023; Kimura et al., 2023). Gu et al. found that MSC-derived EVs induced EMT through the AKT pathway, thereby enhancing the metastasis of GC, however, the key cargoes within these EVs still need to be identified (Gu et al., 2016). Fatty acid oxidation (FAO) in stromal cell metabolic reprogramming plays an important role in tumor metastasis. EVs carrying CD44 derived from GC cells with lymph node metastasis increased FAO activity in Bone marrow-derived MSCs (BM-MSCs) by activating the ERK/PPARγ/CPT1A pathway, resulting in the secretion of IL-8 and STC1, and promoting lymph node metastasis (Huang et al., 2023b). In addition, Mao et al. reported that MSC-derived EVs carrying LINC01559 activated the PI3K/AKT pathway to enhance the migration of GC cells (Wang et al., 2020). EVs secreted by p53-deficient BM-MSCs can transport ubiquitin protein ligase E3 component n-recognin 2 to GC cells and p53 wild-type BM-MSCs, to reprogram cells in the TME and promote GC metastasis by activating the Wnt/β-catenin pathway, but its underlying mechanisms have not been elucidated yet (Mao et al., 2017). MSC-derived EVs can promote GC metastasis, and can also inhibit it. You et al. generated EVs-lipocalin-type prostaglandin D2 synthase (L-PGDS) by transfecting MSCs with an adenovirus encoding L-PGDS. EVs-L-PGDS inhibited the migration and invasion of GC cells and induced apoptosis (You et al., 2022).

## 3 EVs influence the metastasis of GC by modulating immune responses

**FIGURE 3:**
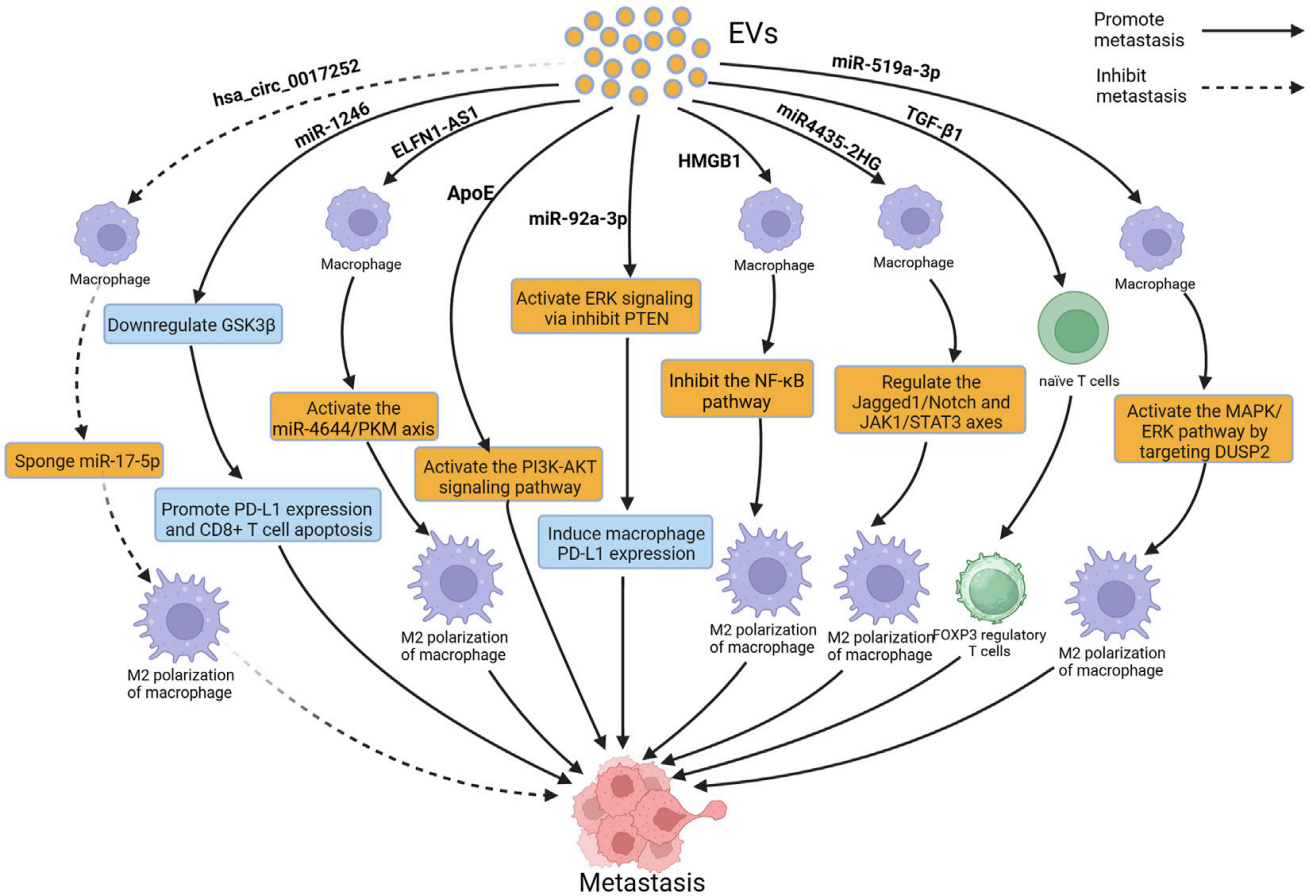
Extracellular vesicles (EVs) can influence gastric cancer (GC) metastasis by modulating immune cells such as macrophages and T cells. Figure was created with BioRender.com.

The process of tumor metastasis is intricately linked to the body’s immune system. To metastasize, tumor cells must evade immune surveillance and escape from the killing mechanisms of the seeding organs (Fan et al., 2020). Immune cells such as macrophages, neutrophils, and T cells can recognize and eliminate tumor cells, preventing or delaying further tumor spread. Additionally, immune cells can produce cytokines and chemokines that regulate the TME and influence the invasive and metastatic abilities of tumor cells (Khan et al., 2023). EVs participate in cell communication by transporting and delivering bioactive substances such as nucleic acids and proteins, to regulate the function of immune cells and influence tumor metastasis (Joyce et al., 2016; Zhang et al., 2020). Figure 3 depicts the relevant mechanisms by which EVs modulate the immune system to influence GC metastasis.

Macrophages are one of the most important components of the immune system. They exist in two subtypes: M1, which inhibits tumor development, and M2, which promotes tumor development. M2 macrophages can alter the TME, promoting the invasion and migration of tumor cells and providing support for tumor cells metastasis (Chen et al., 2023a; Liu et al., 2023). Increasing evidence suggests that EVs can interact with macrophages, induce M2 polarization of macrophages, and promote the metastasis of GC (Ma et al., 2023a). A study reported that EVs derived from GC induced M2 polarization of macrophages through the STAT3 pathway; M2-polarized macrophages secrete IL-6 to promote the migration of GC cells, leading to peritoneal metastasis of GC, but the cargoes that play a key role in these EVs are still unclear (Ito et al., 2021). Li et al. reported that miR4435-2HG from GC-derived EVs promoted M2 polarization in macrophages through the Jagged1/Notch and JAK1/STAT3 axis, leading to the metastasis of GC (Li et al., 2022). In the study by Qiu et al., miR-519a-3p from GC-derived EVs was found to accumulate mainly in the liver, and EVs enriched with miR-519a-3p stimulated the MAPK/ERK pathway by targeting DUSP2, leading to M2 polarization of macrophages; M2 polarized macrophages accelerated liver metastasis of GC by promoting the formation of pre-metastatic niches and by inducing angiogenesis in the liver. Interestingly, phosphorylation of STAT3 has also been shown to be a key factor in macrophage M2 polarization. However, whether DUSP2 can also promote M2 polarization in macrophages by regulating STAT3 phosphorylation still requires further investigation (Qiu et al., 2022). Similarly, Gu et al. reported that miR-92a-3p from GC-derived EVs accumulated in the lungs. These EVs activated the ERK signaling pathway, induced immune-suppressive phenotypic differentiation of macrophages, increased PD-L1 expression, and promoted lung metastasis of GC. Furthermore, inhibition of the ERK signaling pathway with PD98059 significantly reduced PD-L1 expression in macrophages and inhibited the colonization of GC cells in the lungs (Gu et al., 2023). EVs can promote M2 polarization of macrophages, and can also inhibit M2 polarization. A previous study reported that hsa_circ_0017252 from GC-derived EVs effectively inhibited M2 polarization of macrophages, thereby suppressing the invasion and migration of GC cells (Song et al., 2022). In addition to EVs derived from GC, EVs derived from macrophages can also affect GC metastasis. A study reported that EVs derived from M2 macrophages enhanced the expression of PD-L1 through the P38MAPK pathway, leading to immune escape and promoting the metastasis of GC, however, the study failed to identify the key cargoes in EVs and did not perform more in-depth functional inhibition analysis (Wang et al., 2021a). Apolipoprotein E (ApoE) is a protein with high specificity found in EVs derived from M2-polarized macrophages. EVs secreted by M2 tumor-associated macrophages transfer functional ApoE to GC cells, resulting in activation of the PI3K-AKT signaling pathway and remodeling of the cell cytoskeleton to support migration, thereby promoting the metastasis of GC both *in vitro* and *in vivo* (Zheng et al., 2018).

In addition to macrophages, neutrophils and T lymphocytes are also important components of the immune system, and increasing evidence suggests that EVs can affect GC metastasis by acting on neutrophils and T lymphocytes. Transforming growth factor-β1 (TGF-β1) is an immunosuppressive cytokine produced by immune and tumor cells. Yen et al. reported that overexpression of TGF-β1 in EVs from GC patients was associated with lymph node metastasis, and further research has revealed that TGF-β1 in EVs converted naive T cells into FOXP3 Treg cells *in vitro*, allowing tumor cells to regulate immune surveillance, leading to lymph node metastasis of GC (Yen et al., 2017). EVs derived from GC cells are mainly absorbed by macrophages and NK cells in the lungs and implanted in the lungs, so these EVs can alter the gene expression and cytokine secretion levels of CD8 T cells, inducing apoptosis of CD8 T cells. Prolonged exposure to GC-derived EVs leads to the formation of an immunosuppressive TME in the lungs of mice, resulting in a reduction of CD8 T cells, which promotes lung metastasis of GC. Further research is needed to identify the key cargoes that play a role in these EVs (Liu et al., 2020). Zhang et al. reported that EVs derived from GC cells induced N2 polarization of neutrophils by the HMGB1/TLR4/NF-κB signaling pathway, promoting autophagy and tumor activation, and N2-polarized neutrophils, in turn, promoted the metastasis of GC (Zhang et al., 2018a). In addition, Lu et al. discovered the overexpression of miR-1246 in GC-derived EVs. Mouse experiments have shown that the overexpressed miR-1246 can promote lymphangiogenesis and lymph node metastasis *in vivo*. Further research revealed that miR-1246 promotes lymphangiogenesis and lymph node metastasis by inhibiting GSK3β to enhance PD-L1 expression and induce CD8^+^ T cell apoptosis (Lu et al., 2023).

## 4 EVs affect the metastasis of GC through EMT and MMT

### 4.1 EVs regulate the metastasis of GC by modulating EMT

**FIGURE 4:**
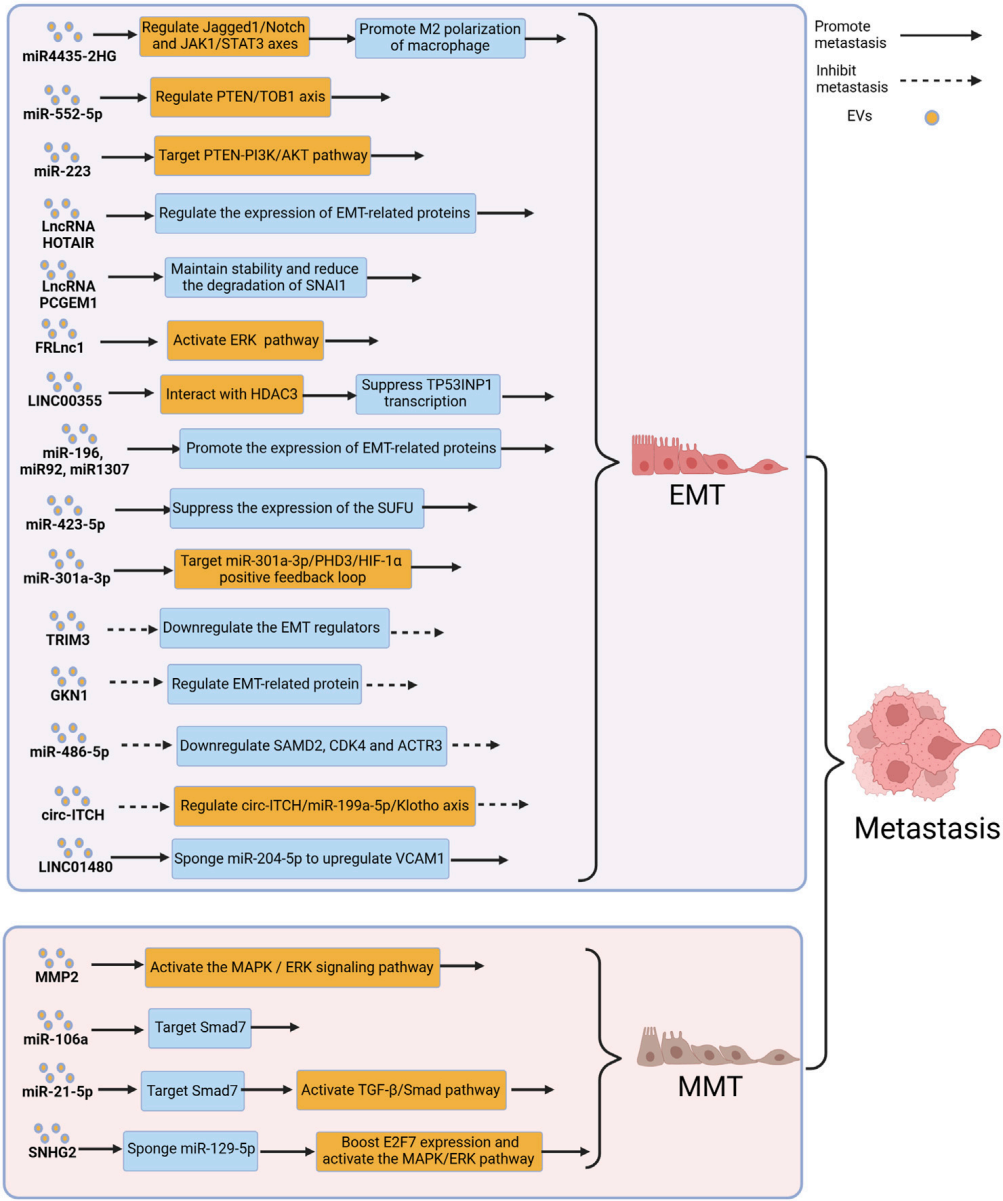
Extracellular vesicles (EVs) can influence gastric cancer (GC) metastasis by modulating the processes of epithelial–mesenchymal transition (EMT) and mesothelial–mesenchymal transition (MMT). Figure was created with BioRender.com.

EMT is considered a driving force for tumor cells metastasis, and is a significant contributor to cancer recurrence and metastasis (Zheng et al., 2020; Zhu et al., 2022b). The loss of epithelial cell markers (such as E-cadherin) and the acquisition of mesenchymal cell markers (such as N-cadherin and vimentin) are two key steps in EMT (Kimura et al., 2023). During EMT, tumor cells lose polarity of epithelial cells and acquire mesenchymal-like motility, which enhances their invasive and migratory abilities, which is considered as the initial stage of tumor metastasis (Maharati and Moghbeli, 2023). EVs can carry various bioactive molecules, including proteins, lipids, and nucleic acids, that can regulate EMT by modulating gene expression, activating related signaling pathways, and altering the TME, thereby influencing the metastatic ability of GC (Pan et al., 2017; Piao et al., 2021; Zhang et al., 2023a; Chen et al., 2023b). Figure 4 depicts the relevant mechanisms by which EVs modulate EMT to influence GC metastasis.

Forkhead box protein M1 (FOXM1) is an oncogene involved in regulating tumor growth and metastasis. A study reported that FOXM1-regulated long non-coding RNA (FRLnc1) was significantly upregulated in serum EVs of GC patients, and a significant association was found between FRLnc1 expression in EVs and GC metastasis. Further research revealed that FRLnc1 overexpression in EVs enhanced the activation of the ERK pathway, downregulated E-cadherin, and upregulated Slug and N-cadherin, promoting EMT in GC cells and facilitating GC metastasis. However, further *in vivo* experiments are needed to confirm this finding. In addition, the specific molecular mechanisms by which FRLnc1 functions in GC metastasis also need to be further studied (Zhang et al., 2021a). Zhao et al. reported that the levels of LINC00355 in plasma EVs of GC patients were significantly higher, when compared with healthy patients, and LINC00355 was found to promote the metastasis of GC cells. Further research revealed that EVs-derived LINC00355 recruited HDAC3 to inhibit the expression of tumor protein 53-induced nuclear protein 1, thereby promoting EMT and resulting in the metastasis of GC (Zhao et al., 2023). Hu et al. reported that EVs derived from malignant ascites of GC patients promoted EMT signaling in GC cells and in a mouse peritoneal tumor model, leading to peritoneal dissemination of the tumor. Moreover, in a mouse peritoneal tumor model, administration of malignant ascites-derived EVs resulted in a significantly reduced median survival, when compared with the control group, however, the molecular mechanisms by which EVs promote peritoneal metastasis of GC has still not been fully understood (Hu et al., 2019). Similarly, Yang et al. found a significant correlation between the elevated expression levels of miR-423-5p in serum EVs of GC patients and lymph node metastasis. Further research revealed that miR-423-5p can suppress the expression of the suppressor of fused protein (SUFU), thereby promoting EMT to facilitate lymph node metastasis in GC (Yang et al., 2018). Hypoxia is a typical characteristic of the TME, and a hypoxic TME leads to changes in tumor features such as angiogenesis, reprogramming of energy metabolism, and immune evasion, resulting in tumor progression (Bristow and Hill, 2008; Jain, 2014; Palazon et al., 2014). Xia et al. reported that in the hypoxic TME, hypoxia-inducible factors 1α increased the release of miR-301a-3p from GC-derived EVs, and treatment of GC cells with these EVs resulted in upregulation of mesenchymal cell markers (N-cadherin and vimentin) and downregulation of epithelial cell markers (E-cadherin), indicating that these EVs promoted tumor metastasis by inducing EMT in GC cells (Xia et al., 2020). An additional study reported that reducing GC-derived EVs altered the molecular mechanisms associated with EMT signaling pathway in GC cells, thereby reducing GC metastasis, particularly peritoneal metastasis, which provided a new direction for the treatment of GC patients (Shibamoto et al., 2022).

Not all EVs promote EMT and contribute to GC metastasis. Tripartite motif-containing 3 (TRIM3) is a key regulator of tumor cell development (Hatakeyama, 2011; Cambiaghi et al., 2012). Compared to healthy patients, GC patients have decreased levels of TRIM3 protein in serum EVs, and knockdown of TRIM3 in EVs from GC patients can alter the expression of EMT-related factors and promote GC metastasis. Conversely, *in vivo* studies have shown that overexpression of TRIM3 in EVs can inhibit EMT and suppress GC metastasis (Fu et al., 2018). Gastric intrinsic factor 1 (GKN1) is a gastric-specific tumor suppressor that maintains mucosal integrity and regulates cell differentiation (Yoon et al., 2014; Xing et al., 2015; Yoon et al., 2018). When GC cells were co-cultured with EVs rich in GKN1, the expression of E-cadherin in GC cells increased, while the expression of proteins such as N-cadherin significantly decreased, suggesting that GKN1 derived from EVs inhibited GC metastasis by suppressing EMT (Yoon et al., 2020). Studies have also shown that biologically active substances such as miR-486-5p and circ-ITCH delivered by EVs could inhibit the metastasis of GC by suppressing EMT (Wang et al., 2021b; Lin et al., 2021).

### 4.2 EVs regulate the metastasis of GC by modulating MMT

MMT refers to the process in which mesothelial cells acquire characteristics of mesenchymal cells, allowing them to gain increased invasiveness (Nakamura et al., 2015). The peritoneum is composed of a monolayer of flat mesothelial cells and a thin layer of submesothelial connective tissue, and the cohesive mesothelial layer, when undamaged, acts as the primary defense against tumor attachment (Mutsaers and Wilkosz, 2007). During MMT, as a consequence of the dissociation from each other within the monolayer, mesothelial cells lose their apical-basal polarity and undergo reorganization of their actin cytoskeleton. In addition, continuous submesothelial connective tissue is also disrupted (Yáñez-Mó et al., 2003; Rynne-Vidal et al., 2015). MMT typically occurs in the early stages of tumor cell peritoneal metastasis, through MMT, the peritoneum can form a pre-metastatic niche, which facilitates the adhesion and colonization of tumor cells (Sandoval et al., 2013). Several studies have therefore reported that EVs could mediate MMT and promote tumor metastasis (Zhu et al., 2020; Gao et al., 2021b; Pascual-Antón et al., 2021). Figure 4 depicts the relevant mechanisms by which EVs modulate MMT to influence GC metastasis.

Deng et al. reported that matrix metalloproteinase 2 in EVs could induce MMT and promote peritoneal metastasis of GC by activating the ERK pathway and increasing the expression of mesenchymal markers, such as vimentin and fibronectin in GC cells (Deng et al., 2017). Previous studies reported that miR-106a was significantly upregulated in GC and may be associated with GC development (Wang et al., 2013; Espinosa-Parrilla et al., 2014). Zhu et al. conducted related studies and found that miR-106a was overexpressed in GC-derived EVs. Stimulation with miR-106a suppressed the expression of Smad7, leading to increased expression of α-SMA and fibronectin in mesothelial cells, to promote MMT and facilitate peritoneal metastasis of GC (Zhu et al., 2022c). Li et al. also found that GC-derived EVs carrying miR-21-5p induced MMT in GC cells through the TGFβ/Smad pathway, resulting in peritoneal metastasis of GC (Li et al., 2018). An additional study reported that the long non-coding RNA small nucleolar RNA host gene 12 (SNHG12) promoted peritoneal metastasis of GC, and the levels of SNHG12 expression in EVs derived from GC patients with peritoneal metastasis were significantly elevated, when compared with those without peritoneal metastasis. GC-derived EVs could deliver SNHG12 to human peritoneal mesothelial cells, inducing MMT and further promoting peritoneal metastasis of GC, and further studies showed that SNHG12 promoted peritoneal metastasis through the miR-129-5p/E2F7/MAPK/ERK axis (Zhang et al., 2022a).

## 5 EVs influence GC metastasis by regulating angiogenesis

**FIGURE 5:**
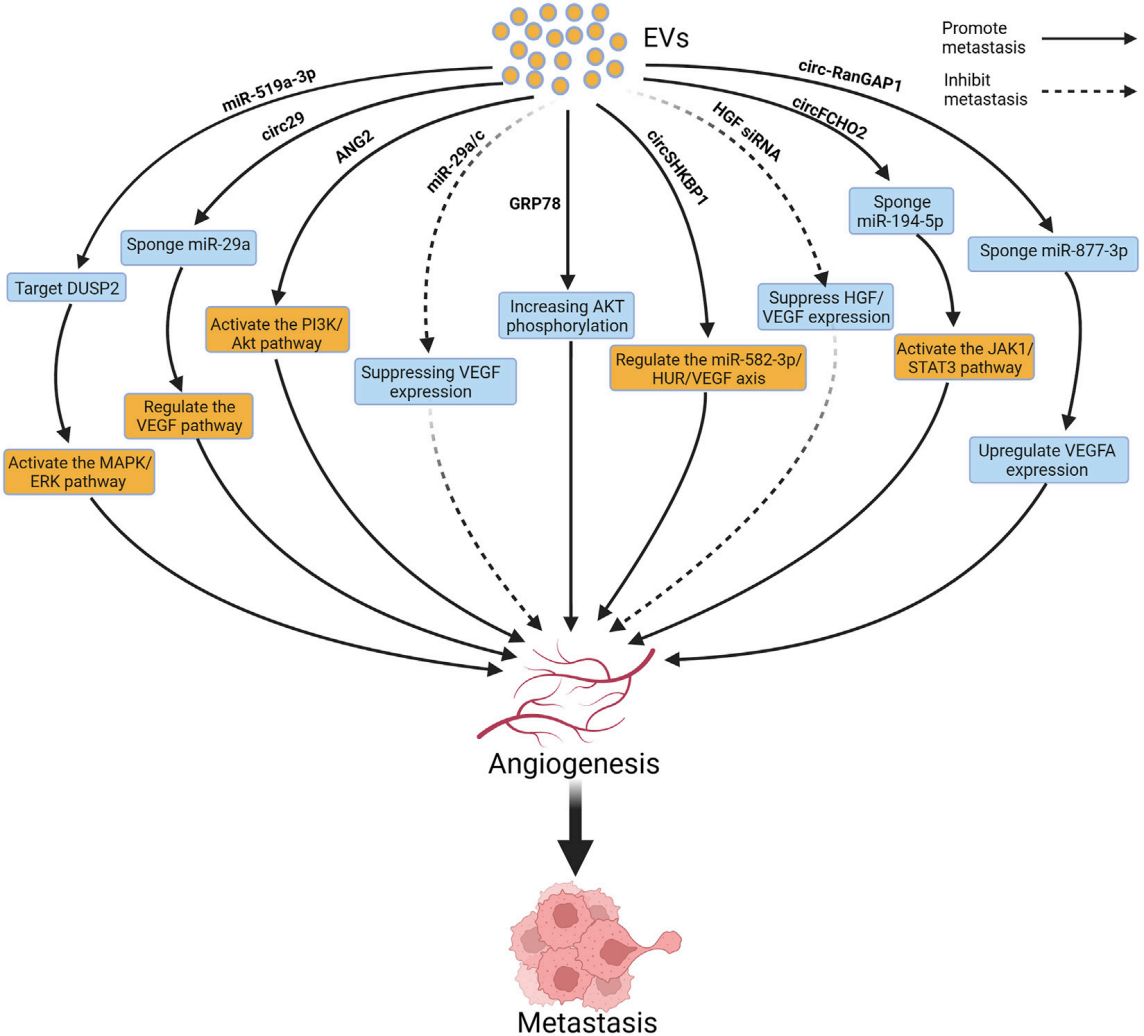
Extracellular vesicles (EVs) can influence gastric cancer (GC) metastasis by inducing angiogenesis. Figure was created with BioRender.com.

Tumor growth and metastasis rely on blood vessels to provide sufficient oxygen and nutrients. Neoangiogenesis provides a new blood supply to tumors, allowing tumor cells to obtain more oxygen and nutrients. It also provides a pathway for tumor cell metastasis using newly formed blood vessels; tumor cells can enter the bloodstream or lymphatic system and spread to distant organs and tissues (Nowosad et al., 2023; Yao and Zeng, 2023). Previous studies reported that EVs delivered various bioactive substances (such as miR-29, miR-10a-5p, circ29, and GRP78) to promote the expression of vascular endothelial growth factor (VEGF), thereby promoting angiogenesis (Zhang et al., 2016; Li et al., 2021; Zhu et al., 2022d; Iha et al., 2022). In addition, EVs increase vascular permeability, making it easier for tumor cells to cross the endothelial barrier of blood vessels to provide increased opportunities for tumor cell metastasis (Dou et al., 2021). Figure 5 depicts the relevant mechanisms by which EVs regulate angiogenesis to influence GC metastasis.

The expression of angiopoietin-2 (ANG2) is significantly increased in GC-derived EVs. ANG2 can activate the PI3K/AKT signaling pathway to regulate angiogenesis. Moreover, ANG2 is upregulated in GC omental metastatic samples, indicating that ANG2 may promote GC metastasis by regulating angiogenesis (Kalfon et al., 2022). The circ-RanGAP1 is overexpressed in the EVs from plasma of GC patients and GC tissues, and elevated expression of circ-RanGAP1 is strongly correlated with the prognosis of patients with GC. In addition, circ-RanGAP1 can induce VEGFA expression by sponging miR-877-3p, to promote angiogenesis and thereby facilitate GC metastasis. Further experimental and clinical studies are needed in the future to confirm whether treatments targeting circ-RanGAP1 can be applied in clinical settings (Lu et al., 2020). EVs enriched with circSHKBP1 are upregulated in GC patients, and the level of circSHKBP1 in EVs significantly decreases after GC surgery. CircSHKBP1 promotes VEGF secretion and induces its expression by sponging miR-582-3p, to promote angiogenesis, leading to lung metastasis of GC (Xie et al., 2020). The expression of miR-519a-3p in serum-derived EVs is significantly elevated in GC patients with liver metastasis (LM), when compared with those without LM. EVs derived from GC with overexpression of miR-519a-3p primarily accumulate in the liver, and induce M2 polarization of macrophages by targeting DUSP2 and activating the MAPK/ERK pathway, while M2-polarized macrophages accelerate GC-LM by inducing angiogenesis (Qiu et al., 2022). CircFCHO2 enhances the progression of GC by activating the JAK1/STAT3 signaling pathway by sponging miR-194-5p, and silencing circFCHO2 weakens angiogenesis and cancer stem cell characteristics in GC cells. In addition, *in vivo* studies have shown that silencing circFCHO2 inhibited lung metastasis of GC (Zhang et al., 2022b). A similar study reported that EVs containing hepatocyte growth factor siRNA administered through tail vein injection in mice effectively inhibited tumor angiogenesis (Zhang et al., 2018b). EVs promote angiogenesis and increase vascular permeability. Wang et al. reported that plasma levels of EVs in mice with GC and lung metastasis were significantly higher than those without lung metastasis, indicating that EVs may promote GC metastasis. Further studies showed that EVs activated endothelial cells and induced cytoskeletal reorganization through a dynamin-dependent pathway, disrupting the endothelial barrier and inducing vascular leakage, leading to lung metastasis of GC. However, the specific mechanisms of action of these EVs and their key cargoes are still unclear (Wang et al., 2022b).

## 6 Organotropism

With the introduction of Stephen Paget’s “seed and soil” theory, it has been recognized that tumor metastasis is not random but rather a selective process that targets specific organs (Akhtar et al., 2019). This phenomenon is known as organotropism in tumor metastasis. GC also exhibits organotropism during the metastatic process, where GC cells possess the ability to selectively migrate to certain organs such as the lungs, liver, and peritoneum (Urabe et al., 2021; He et al., 2023). This selective metastasis may be attributed to the affinity of EVs released by GC cells for specific recipient cells. Hoshino et al. discovered that EVs containing integrins can specifically bind to recipient cells, thereby forming pre-metastatic niches and promoting metastasis organotropism in GC (Hoshino et al., 2015). Additionally, Qiu et al. found that EVs rich in miR-519a-3p can induce M2-like polarization of macrophages through the MAPK/ERK pathway, and M2-like polarized macrophages facilitate the formation of pre-metastatic niches in the liver, thereby inducing liver-specific metastasis in GC (Qiu et al., 2022). Similarly, studies have indicated that EVs can promote metastasis organotropism of GC to organs such as the lungs and peritoneum (Li et al., 2018; Zhang et al., 2022a).

## 7 Perspectives and future directions

EVs serve as important intercellular communication mediators and have great potential in the field of oncology. EVs carry a diverse range of biomolecules that can not only influence tumor growth and metastasis but also serve as tumor markers for cancer diagnosis (Su et al., 2023). Analyzing biomarkers within EVs released by tumor cells therefore facilitates noninvasive tumor diagnosis and monitoring, compared to conventional diagnostic methods, it has the advantages of being rapid, cost-effective, and highly specific (Zhang et al., 2022c; Ma et al., 2023b). For example, the detection of urinary 3-gene expression levels can differentiate between high-grade and low-grade prostate cancer, as well as benign prostate diseases (McKiernan et al., 2016). Additionally, EVs can serve as carriers for drug or small interfering RNA (siRNA) delivery, they can be engineered to possess specific targeting and drug delivery functions. By encapsulating drugs or siRNA within EVs, their stability and bioavailability can be enhanced, while reducing their side effects (Kimura et al., 2023; Zeng et al., 2023). For example, Yu et al. discovered that encapsulating VEGFR2 siRNA within EVs for the treatment of lung metastasis in mice with osteosarcoma is a more efficient and less toxic therapeutic approach (Yu et al., 2023). Similarly, EVs have demonstrated great potential as efficient delivery vehicles for drugs such as paclitaxel and gemcitabine, highlighting their significant role in tumor therapy (Saari et al., 2015; Li et al., 2020b).

However, there are still some limitations in the current application of EVs in tumors. First, obtaining a sufficient quantity and quality of EV samples remains a challenge. The current sample sources primarily include body fluids such as saliva, urine, and blood, but the content of EVs in these fluids is low, and there are many nonspecific EVs present, limiting the accuracy and reliability of their applications (Zhang et al., 2022c). Furthermore, EVs are highly complex particles whose composition and function are influenced by various factors such as cell type, state, and the environment. Accurate identification, isolation, and purification of EVs remain challenging and require further development of technologies and methods (Wu et al., 2021). The emerging methods of EVs separation based on size and charge, as well as single EV analysis techniques in recent years, may help to address these issues (Ko et al., 2021; Zhang et al., 2023b; Chen et al., 2023c). Third, despite extensive studies on the roles of EVs in tumors, the specific mechanisms of action of EVs, especially the signaling pathways, are still not fully understood. In future studies, it is necessary to further explore the specific molecular mechanisms by which EVs affect GC metastasis (Wang et al., 2022b). Finally, although numerous studies have reported the mechanisms of EVs during tumor metastasis, current research is mainly limited to cell and animal experiments, how to apply these findings to the clinic will be one of the key focuses of future work (Kalluri and McAndrews, 2023).

## 8 Conclusion

This review focuses on summarizing the biological roles of EVs in GC metastasis from the following aspects. Firstly, EVs can affect the cell interactions and signal transduction in TME by releasing bioactive molecules, thus regulating the process of GC metastasis (Zhang et al., 2021b; Li et al., 2023b). Secondly, the molecules released by EVs, such as metastasis-related proteins and non-coding RNAs, can regulate the EMT and MMT of GC cells, enhance the metastatic ability of GC (Deng et al., 2017; Babaei et al., 2022; Ang et al., 2023). In addition, immune regulatory molecules contained in EVs can regulate tumor immune escape and immune suppression, thus affecting the immune recognition and clearance of GC cells and affecting their metastasis (Kiya et al., 2023; Yi et al., 2023). Finally, the bioactive molecules in EVs can regulate the expression of vascular growth factors, thereby affecting the formation of new blood vessels. The newly formed blood vessels not only increase the blood supply and nutrition of GC cells but also enhance their metastatic ability (Wang et al., 2022b; Qiu et al., 2022). It is also worth noting that tumor metastasis is a multi-step and complex process, and EVs often interact through previously mentioned multiple pathways to influence GC metastasis, so a thorough investigation of the mechanisms of EVs in GC metastasis is crucial for GC. Although a growing body of studies has revealed the biological roles of EVs in GC metastasis, challenges and limitations still exist. With the development of technologies and research, our understanding and application of EVs will continue to improve, providing more options and opportunities for diagnosing and treating GC.
